# miR-26b-5p promotes osteogenesis of bone mesenchymal stem cells via suppressing FGF21

**DOI:** 10.1097/MD.0000000000035333

**Published:** 2023-09-22

**Authors:** Bin Wang, Zhenhui Li, Caiyuan Mai, Penglin Mou, Lei Pan

**Affiliations:** a Department of Orthopaedics, Foshan Sanshui District People’s Hospital, Guang Hai Rode, Foshan, China; b Department of Intervention Department, Guangzhou Red Cross Hospital, Tong Fu Rode, Guangzhou, China; c Department of Obstetrics, Guangdong Women and Children’s Hospital, Guang Yuan Road, Guangzhou, China; d Department of Orthopaedics, Guangzhou Xinhai Hospital, Xin Gang Road, Guangzhou, China.

**Keywords:** BMSCs, FGF21, miR-26b-5p, postmenopausal osteoporosis

## Abstract

**Background:**

miR-26b-5p actively participates in the osteogenic differentiation of bone mesenchymal stem cells (BMSCs). The database showed that fibroblast growth factor (FGF)-21 is a potential binding site of miR-26b-5p. This study aimed to investigate the molecular osteogenic mechanisms of miR-26b-5p targeting FGF21.

**Methods:**

Bone marrow was aspirated from the anterior superior iliac spine during bone marrow puncture. BMSCs were used to establish an in vitro cell model, and BMSCs markers were analyzed by flow cytometry. miR-26b-5p were overexpressed for 48 hours, and then placed in an osteogenic induction medium for osteogenic induction culture, the expression of RNA was detected using RT-qPCR. On day 7 of induction, RT-qPCR was used to measure Runx2, Osterix (Osx), and target gene FGF21 expression levels in each group. RT-qPCR, the dual-luciferase reporter gene system and western blot were used to verify that FGF21 was a direct target of miR-26b-5p.

**Results:**

BMSCs were identified according to the antigenic characteristics. miR-26b-5p expression was significantly upregulated after the expression of miR-26b-5p mimics, and FGF21 expression was downregulated; in miR-26b-5p inhibitor, the opposite results were revealed. After overexpression of miR-26b-5p, the alkaline phosphatase activity and nodules of Alizarin red S in the culture medium was increased; the opposite results were revealed in miR-26b-5p inhibitor. The expressions of Runx2 and Osx in the miR-26b-5p group were also significantly higher; in the miR-26b-5p inhibitor group, the opposite results were revealed. Luciferase reporter assays demonstrated that FGF21 was a direct target of miR-26b-5p. The western blotting analysis showed that FGF21 expression was significantly downregulated in the miR-26b-5p overexpressed group. Finally, the expressions of the characteristic osteogenic factors in the miR-26b-5p control + FGF21 group was significantly lower, but then increased significantly in the miR-26b-5p mimics + FGF21 group; the expressions of the characteristic osteogenic factors in the miR-26b-5p control + si-FGF21 group was significantly higher.

**Conclusions:**

miR-26b-5p can regulate the osteogenic differentiation of BMSCs and participate in PMOP pathogenesis via suppressing FGF21.

## 1. Introduction

Osteoporosis is a common metabolic bone disease characterized by decreased bone mass and strength.^[1]^ More than 70 million people in China reportedly have osteoporosis, and the prevalence rate in women over 50 years old is 20.7%.^[2]^ It has been shown that the decrease of estrogen levels after menopause leads to endocrine disorders, which further affect calcium absorption and metabolism, leading to bone resorption, bone destruction and low back pain. The incidence of osteoporosis in postmenopausal women is reportedly 2 to 3 times that of non-menopausal women.^[3]^ Most importantly, osteoporosis affects patient quality of life and is a major socio-economic health problem.^[4]^

Bone homeostasis is maintained by a balance between bone resorption by osteoclasts and bone formation by osteoblasts to maintain normal structure and function.^[5]^ Abnormal osteogenesis occurs when this balance is disrupted, leading to osteoporosis or metabolic bone diseases.^[6]^ microRNAs (miRNAs) are small noncoding single-stranded RNA molecules that have been extensively studied in tumors, bone metabolism and other research areas.^[7]^ miRNAs mainly bind to the 3′ noncoding region of target genes and negatively regulate expression of target factors, leading to degradation or cessation of the translation process, thus affecting cell proliferation, migration, and apoptosis.^[8]^ In recent years, the biological role of miRNA in bone mesenchymal stem cells (BMSCs), osteoblasts and osteoclasts has attracted mounting attention. miR-26b-5p can promoted osteogenic differentiation.^[9]^ Studies have documented that miR-26b-5p is a positive regulator of goat intramuscular preadipocyte via targeting fibroblast growth factor 21 (FGF21).^[10]^ So we hypothesized that FGF21 could be involved in the regulation of osteogenesis.

Fibroblast growth factor (FGF) 21, a special member of FGF superfamily, has been proven to have pleiotropic metabolic effects and many potential therapeutic action in various metabolic disorders.^[11]^ Study showed that FGF21 in high glucose environment could inhibit the osteogenic differentiation of BMSCs.^[12]^ FGF21 might be an efficient endogenous vasoprotective factor for calcification.^[13]^

It remains unclear whether FGF21 can be leveraged as a target of miR-26b-5p to regulate BMSCs differentiation, warranting further studies. This study aimed to investigate the regulatory mechanisms of miR-26b-5p on BMSCs in humans.

## 2. Experiment instruments and reagents

Ultraviolet spectrophotometer, Real-time PCR detection system, flow cytometer, electrophoresis apparatus, and transfer membrane apparatus were purchased from Bio-rad Corporation, Hercules, CA. The Luciferase reporter assay was purchased from Abcam Corporation, Cambridge, England.

The RNA Extraction Kit, reverse transcription Kit, and primers were purchased from Takara Corporation, Beijing, China. The BCA Protein Quantitation Kit was purchased from Doyang Corporation, China. Primary and secondary antibodies used during western blotting were purchased from Abcam Corporation, Cambridge, England. ALP kit, Alizarin red S staining kit, BCIP®/NBT solution, and carriers were purchased from Bio-Rad, Hercules, CA. DMEM high sugar medium and osteogenic differentiation medium were purchased from Sigma Corporation, Ronkonkoma, NY. Fetal bovine serum, pMIR-REPORT plasmid, lipidosomes, Lipofectamine 2000, and 293T cells were purchased from Invitrogen Corporation, Waltham, MA. A plasmid extraction kit was purchased from QIAGEN Corporation, Hilden, Germany. Controls, mimics, inhibitor, si-FGF21, and Trizol reagent were purchased from Jima Corporation, China.

## 3. Methods

This study and all methods were carried out in accordance with relevant guidelines and regulations, they were approved by the Ethics Committee of the Foshan Sanshui District People’s Hospital (Ethical review number: 2019003) in accordance with the Helsinki Declaration of 1975 (revised in 2000). Informed consent was obtained from all individual participants included in the study. Sixteen postmenopausal women were recruited from July 2021 to October 2021. 5ml of bone marrow was aspirated from the anterior superior iliac spine in all patients during bone marrow puncture. Prior to the study, the study participants exhibited no mental, cognitive or mobility-related impairment. A diagnosis of postmenopausal osteoporosis (PMOP) was established with bone mineral density *T*-value ≤ −2.5 measured by dual-energy X-ray absorptiometry. Patients with severe chronic diseases that cause metabolic abnormalities such as secondary PMOP and patients that received drug or hormone therapy within the past year were excluded.

### 3.1. BMSCs culture

5 mL bone marrow was collected in lithium heparin tubes. To establish an in vitro culture system for osteogenic differentiation of BMSCs, the bone marrow was evenly mixed with an equal volume of DMEM culture medium containing antibody. The mixture was centrifuged at room temperature, and the supernatant was removed. The cells were resuspended in a DMEM culture medium. The cell suspension was slowly added to an equal volume Percoll separation solution and centrifuged at room temperature. The middle layer of mononuclear cells was resuspended with culture medium. After full mixing, a suspension containing BMSCs was obtained and cultured in a 5% CO_2_ incubator at 37 °C. The fluid was changed every 24 hours; the cell morphology, size, and distribution were observed under an inverted microscope. After trypsin digestion, the DMEM medium was used to adjust the cell concentration to 2 × 10^5^ and inoculated in cell culture plates and cultured. When the degree of cell fusion reached 70% to 80%, trypsin was added, the cell suspension was collected and inoculated into a culture flask for subculture. Third-generation BMSCs in a logarithmic growth stage were added to an osteogenic induction solution (α-MEM culture medium containing fetal bovine serum, dexamethasone, ascorbic acid and β-sodium glycerophosphate) for 3 weeks. Cells were then collected, and the total RNA was extracted.

### 3.2. Identification of BMSCs

A BMSC suspension of 1 × 10^6^ cells/mL was prepared. The cells were washed twice with cold PBS, centrifuged at 1000 × g for 5 minutes at 4 °C, and resuspended in 100 mL stain buffer. The resuspended cells were incubated with phycoerythrin-labeled primary antibodies against surface markers CD34, CD45, CD73, CD105, and a corresponding isotype control antibody at room temperature, according to the manufacturer’s protocol. The positively stained cells were analyzed by flow cytometry using FlowJo software 8.7.1. BMSCs from passages 3 to 6 were used in the experiments.

### 3.3. Osteogenic differentiation and ALP/Alizarin Red S staining

Cells from passages 3 to 6 of each group were transfected for overexpression and suppression of miR-26b-5p. Alizarin Red S staining was conducted on days 7, 14, 21, and 28 of induction to assess calcium deposit formation. Alkaline phosphatase (ALP) staining was conducted on days 7, 14, and 21 of induction to assess ALP activity.

For ALP staining, cells were fixed with 10% formaldehyde for 15 minutes, rinsed 3 times with deionized water, and treated with the BCIP®/NBT solution for 20 minutes. After washing, the stained cultures were photographed. To measure ALP activity, cell lysates were tested using a commercial ALP assay kit. For Alizarin red S staining, BMSCs were stained with pH 4.2, 0.1% Alizarin red S for 5 minutes, and the images were captured using a scanner. The calcium deposition was dissolved in 10 cetylpyridinium chloride, and the absorbance of the extracts was determined at 570 nm.^[14]^

### 3.4. RT-qPCR

Total RNA was extracted from cultured cells or bone tissues using RNAiso Plus, and cDNA was synthesized using PrimeScript RT Master Mix. Real-time PCR was performed using primers which were synthesized by Thermo Fisher Scientific. The sequences of primers used are as follows: 5′ to 3′: Runx2-F CTCCTACCTGAGCCAGATGACG, Runx2-R GTGTAAGTAAAGGTGGCTGGATAGT; Osx-F CCAAGTGGGTGGTATAGAG, Osx-R GGGATGGTGGGTGTAAGA; FGF21-F GCCTCTAGGTTTCTTTGCC, FGF21-R GACTCCTGGTTGCTCTTGG; β-actin-F TGGCACCCAGCACAATGAA, β-actin-R CTAAGTCATAGTCCGCCTAGAAGCA. The PCR procedure consisted of denaturation at 94 °C for 5 minutes; 30 cycles of denaturation at 94 °C for 30 seconds; annealing at 58 °C for 30 seconds and extension at 72 °C for 40 seconds; extension at 72 °C for 10 minutes. β-Actin was selected as the internal reference. Data were expressed using the comparative CT (2−ΔΔCT) method and normalized to β-actin.

### 3.5. Cell transfection

We purchased miR-26b-5p mimics, the corresponding negative (NC), miR-26b-5p inhibitor and the control. Cells were seeded in twenty-four-well plates at a density of 5 × 10^5^; when the cell fusion degree reached 90%, the culture medium was discarded, and cells were washed twice with PBS buffer and placed in 3 mL of Opti-MEM culture medium in each well. The plasmids were diluted to 20 uM with an Opti-MEM culture medium and transfection reagent. The corresponding groups of RNA were added to the transfection reagent, incubated for 20 minutes, and then added to the culture medium. After 6 hours of culture, it was replaced with an ordinary culture medium, and the expression level was verified after culture for 48 hours. miR-26b-5p was overexpressed and suppressed for 48 hours, and then placed in an osteogenic induction medium for osteogenic induction culture, the expression of RNA was detect using RT-qPCR. Cells from miR-26b-5p group were collected on days 7, 14, and 21 of induction for ALP and on days 7, 14, 21, and 28 of induction for Alizarin red S staining. They were divided into 3 groups: BMSC + miR-26b-5p control group (NC), BMSC + miR-26b-5p overexpression group (mimics), and BMSC + miR-26b-5p suppression group (inhibitor). The expressions of osteogenic specific genes Runx2 and Osx and target factor FGF21 were measured by RT-qPCR on day 7 of osteogenic induction.

### 3.6. Dual-luciferase reporter gene assay

Using TargetScanFish software, we found miR-26b-5p combine with FGF21. We performed a dual-luciferase binding assay to verify the binding site and cloned FGF21 3′-UTR containing the predicted miR-26b-5p binding site in the pMIR-REPORTTM miRNA Expression Reporter vector. The plasmid was named pMIR-FGF21-WT (wild type). A mutated FGF21 reporter gene was created from the mutation-binding site region using a site-directed mutagenesis kit and named PMIR-FGF21-MT (mutant type). For transfection, cells were inoculated in 24-well plates, and 100 ng pMIR-FGF21-WT or pMIR-FGF21-MT fluorescence reporter vector and 50ng miR-26b-5p mimics were co-transfected into 293T cells using Lipofectamine 2000. After 48 hours, luciferase activity was measured with the luciferase assay system according to the manufacturer’s instructions.

### 3.7. Protein extraction and western blotting (WB) analysis

Cell samples were rinsed twice with cold PBS and harvested in the lysis buffer. The lysate was centrifuged at 4 °C/16,000 × g for 30 minutes, and the suspension was collected. Then, the protein content was examined using a BCA kit. Total proteins (20 µg) were separated by 12% (w/v) SDS-PAGE. The proteins were then transferred onto a polyvinylidene fluoride membrane. The membrane was washed and blocked with freshly prepared TBST containing 5% (w/v) nonfat dry milk for 90 minutes at room temperature. The membrane was incubated with antibodies targeting FGF21 and β-actin overnight at 4 °C. After washing 3 times, the membrane was incubated with horseradish peroxidase-conjugated goat anti-rabbit or anti-mouse secondary antibody for 1 hour at room temperature. The membrane was again washed 3 times; then, the protein-antibody complexes were examined using an enhanced chemiluminescent detection reagent. Antibody signals were developed using a Bio-Rad XRS chemiluminescence detection system. Protein band densities were analyzed using Quantity One Software. The mean expression levels of the proteins relative to β-actin were presented.^[14]^ FGF21 was measured by western blotting in the miR-26b-5p overexpression group and in the miR-26b-5p inhibition group.

### 3.8. Statistical analysis

All measurements were repeated 3 times and analyzed using SPSS (v.22.0). Differences between 2 groups were determined by the Student *t*-test, while multigroup comparison was performed by one-way analysis of variance followed by Bonferroni post hoc test. All data were expressed as mean ± standard deviation. A *P*-value <.05 was statistically significant.

## 4. Results

### 4.1. Culture and identification of BMSCs

**Figure 1.**
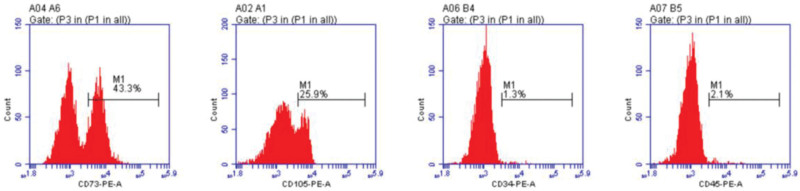
Cell surface marker molecular evidence for identifying circulating BMSCs by flow cytometry. BMSCs = bone mesenchymal stem cells.

The surface markers of BMSCs were analyzed by flow cytometry (Fig. 1). We found that the BMSCs were positive for CD73, CD105 and negative for CD34, CD45. These mesenchymal stem cells were confirmed to be BMSCs, which accords with the antigenic characteristics of mesenchymal stem cells.

### 4.2. Overexpression of miR-26b-5p promoted osteogenic differentiation and miR-26b-5p inhibitor suppress osteogenic differentiation

**Figure 2.**
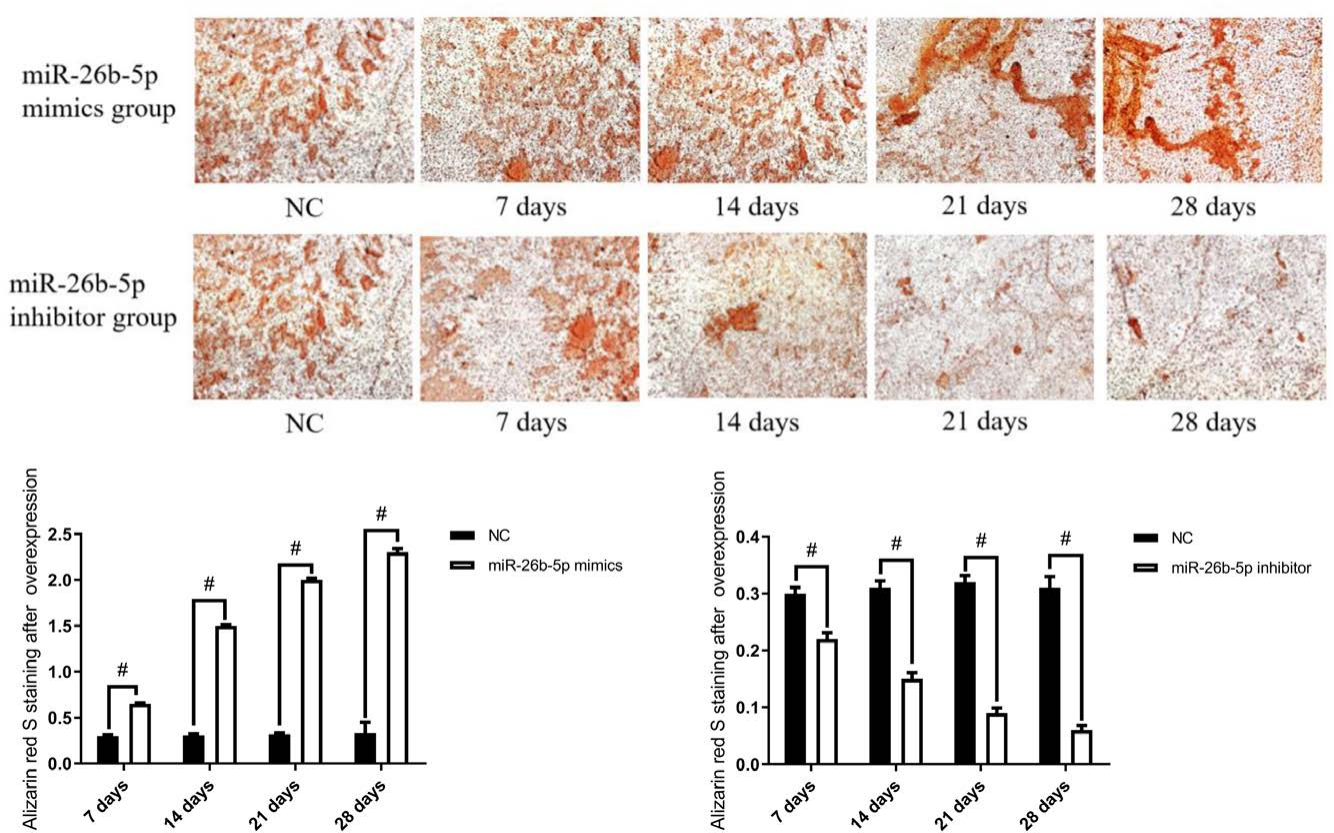
The osteogenic differentiation ability of BMSCs (n = 16) (The scale of electron microscopy: 100 μm). ^#^*P* < .05. BMSCs = bone mesenchymal stem cells.

**Figure 3.**
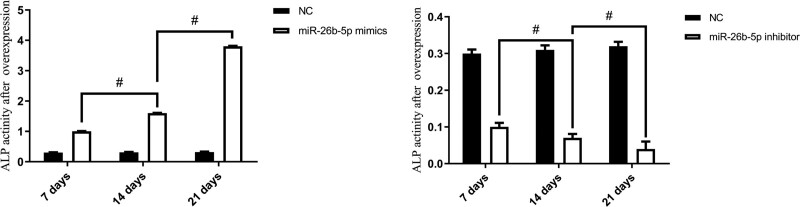
ALP showed that the osteogenic differentiation ability of BMSCs (n = 16). ^#^*P* < .05. ALP: alkaline phosphatase, BMSCs = bone mesenchymal stem cells.

Cells from passages 3 to 6 of each group were transfected for overexpression of miR-26b-5p and suppression of miR-26b-5p. After 48 hours, the culture medium was replaced with an osteogenic induction medium, and ALP was performed on days 7, 14, and 21, Alizarin red S staining experiments were performed on days 7, 14, 21, and 28. The results showed that after miR-26b-5p was overexpressed, the level of ALP in the cell culture medium gradually increased with an increase in induction time, and Alizarin red S staining showed progressively increased calcium nodule deposition. However, in miR-26b-5p inhibitor, the opposite results were revealed (Figs. 2 and 3).

### 4.3. The expressions of miR-26b-5p and FGF21 after the expression of miR-26b-5p mimics and miR-26b-5p inhibitor.

**Figure 4.**
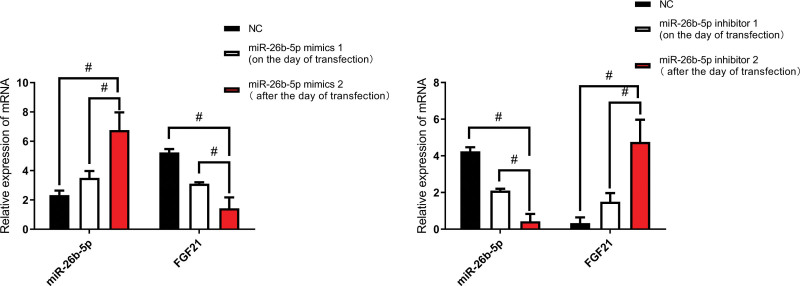
The expression after miR-26b-5p mimics and inhibitor (n = 16). ^#^*P* < .05.

miR-26b-5p expression was significantly increased after the expression of miR-26b-5p mimics; however, FGF21 expression was decreased after miR-26b-5p mimics, the difference is stark. However, in miR-26b-5p inhibitor, the opposite results were revealed (Fig. 4).

### 4.4. Binding site verification of miR-26b-5p and FGF21

**Figure 5.**
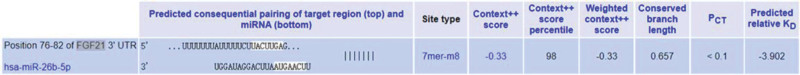
The binding site of mRNA of FGF21 and miR-26b-5p. FGF21: fibroblast growth factor 21.

**Figure 6.**
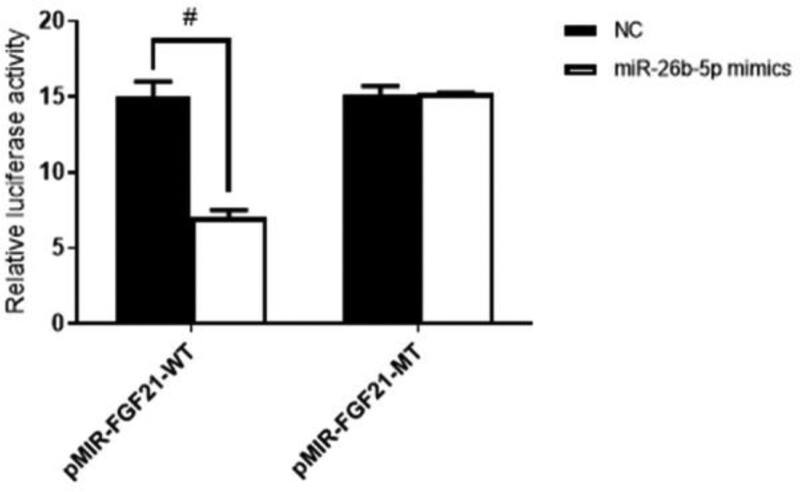
Luciferase reporting experiment (n = 16). ^#^*P* < .05.

A microRNA Target Prediction Database showed that FGF21 is a potential binding site of miR-26b-5p according to the sequence information of the miRNA. The identified binding sites are shown in Figure 5. The luciferase reporter assay showed that miR-26b-5p significantly inhibited the luciferase activity of wild-type (pMIR-FGF21-WT) and did not affect the mutant luciferase reporter gene activity (Fig. 6).

### 4.5. The expressions of Runx2, Osx, and FGF21 in BMSCs were detected by RT-qPCR in miR-26b-5p overexpression and suppression group

**Figure 7.**
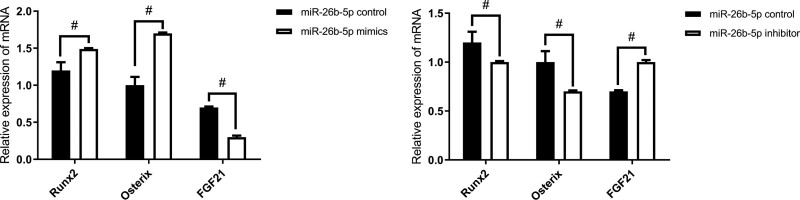
The expressions of Runx2, Osx and FGF21 in BMSCs (n = 16). ^#^*P* < .05. BMSCs = bone mesenchymal stem cells.

On day 7 of induction, cells were collected, and RNAs were extracted. RT-qPCR showed that the expressions of the characteristic osteogenic factors (Runx2, Osx) in the BMSC + miR-26b-5p overexpression group was significantly higher than in the control group. Moreover, target gene FGF21 expression was significantly lower in the BMSC + miR-26b-5p overexpression group than in the control group. However, in the miR-26b-5p inhibitor group, the opposite results were revealed (Fig. 7).

### 4.6. WB analysis of FGF21 expression

**Figure 8.**
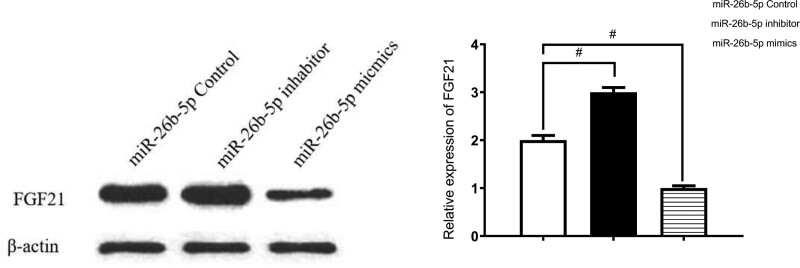
WB detection in BMSCs: expression of FGF21 was negatively correlated with miR-26b-5p expression level. BMSCs = bone mesenchymal stem cells, FGF21 = fibroblast growth factor 21, WB = western blotting.

WB analysis showed that FGF21 expression in 293T cells was significantly decreased in the miR-26b-5p overexpression group (*P* < .05) and was significantly increased in the miR-26b-5p inhibition group (*P* < .05) (Fig. 8).

### 4.7. miR-26b-5p overexpression in BMSCs, the expressions of Runx2 and Osx after FGF21 knockdown

miR-26b-5p overexpression in BMSCs with and without FGF21 knockdown and investigate the expression levels of the osteogenic markers.

**Figure 9.**
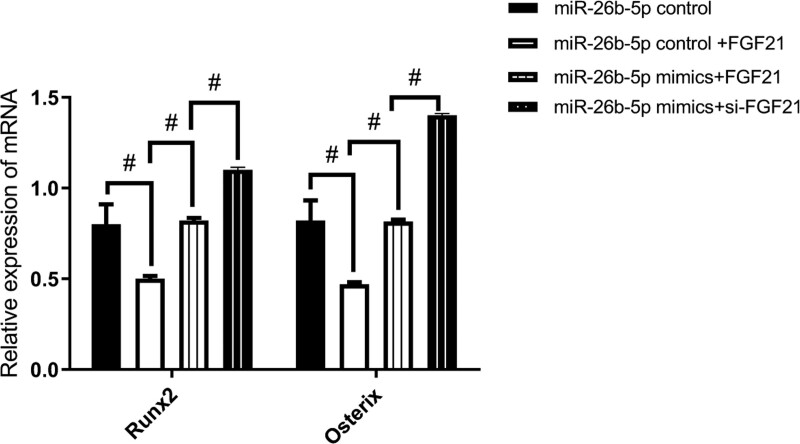
miR-26b-5p overexpression in BMSCs, the expressions of Runx2 and Osx after FGF21 knockdown (n = 16). ^#^*P* < .05. BMSCs = bone mesenchymal stem cells, FGF21 = fibroblast growth factor 21.

It showed that the expressions of the characteristic osteogenic factors (Runx2, Osx) in the miR-26b-5p control + FGF21 group was significantly lower than in the control group, but then increased significantly in the miR-26b-5p mimics + FGF21 group (*P* < .05) (Fig. 9).

It showed that the expressions of the characteristic osteogenic factors (Runx2, Osx) in the miR-26b-5p control + si-FGF21 group was significantly higher (Fig. 9).

These findings suggested that miR-26b-5p overexpression may reverse the suppression effect of FGF21 on osteogenic differentiation-associated protein expression levels; and miR-26b-5p overexpression may enhance the promotive effect of si-FGF21 on osteogenic differentiation-associated protein expression levels.

## 5. Discussion

It is widely acknowledged that PMOP results from the rapid decrease in estrogen levels in women after menopause, and an increase in osteoclasts leads to bone resorption. Interestingly, the osteogenic differentiation ability of BMSCs is weakened, which induces relatively greater bone resorption than bone formation and results in metabolic bone disease.^[13]^ As noncoding single-stranded RNAs with regulatory functions, miRNAs are abundant in BMSCs and affect the proliferation, migration, differentiation, and other biological functions of osteogenic and osteoclast cells.^[10]^ In recent years, the effect of miRNA on BMSCs differentiation, osteoclast and osteoblast differentiation has attracted much attention. miR-26b-5p mimics markedly reduced PTEN, miR-26b-5p overexpression also rescued the inhibitory effect of GAS5 on calcification, and miR-26b-5p promoted osteogenic differentiation.^[9]^

Study found that FGF21 directly promoted RANKL-induced osteoclastogenesis from bone marrow macrophages, as well as promoted adipogenesis while concomitantly inhibiting osteogenesis of bone marrow mesenchymal stem cells (BMMSCs).^[11]^ A Study reveal that skeletal fragility may be an undesirable consequence of chronic FGF21 administration.^[15]^

Conversely, augmentation of miR-100-5p using a specific agomir in OVX-operated mice decreased the levels of FGF21 in the serum and liver, and prevented osteoclastogenesis and bone loss. A study revealed that FGF21 may be a signal molecule associated with the mechanism of liver-bone endocrine metabolism and may be targeted by miR-100-5p. In addition, miR-100-5p may serve an important role in protecting against OVX-induced osteoporosis.^[16]^ Studies have documented that miR-26b-5p is a positive regulator of goat intramuscular preadipocyte via targeting FGF21.^[10]^

However, few studies explored whether the regulatory role of miR-26b-5p in human BMSCs was mediated by FGF21. In the present study, we first identified BMSCs and found they accord with the antigenic characteristics. The results of Alizarin red S staining and ALP in the cell culture medium gradually increased showed that the osteogenic differentiation ability of BMSCs was significantly increased after overexpression of miR-26b-5p; in miR-26b-5p inhibitor, the opposite results were revealed. This suggests that miR-26b-5p can enhance osteogenic differentiation.

miR-26b-5p expression was significantly increased after the expression of miR-26b-5p mimics while FGF21 expression was decreased; the opposite results were revealed in miR-26b-5p inhibitor. This reflects the inhibitory relationship between miR-26b-5p and FGF21. Using a target prediction website, target gene FGF21 was predicted to be the potential binding site of miR-26b-5p. The binding sequences of the wild and mutant FGF21 gene were synthesized. The luciferase reporter assay showed that miR-26b-5p significantly inhibited the luciferase activity of wild-type (pMIR-FGF21-WT) and did not affect the mutant luciferase reporter gene activity, indicating the miR-26b-5p mimics were able to bind to 3′-UTR of wild-type FGF21 mRNA, and that FGF21 is the target gene of miR-26b-5p.

On day 7 of osteogenic induction, Runx2 and Osx expressions were higher in the BMSC + miR-26b-5p overexpression group than in the control group. FGF21 expression was significantly lower in the BMSC + miR-26b-5p overexpression group than in the control group; The opposite results were revealed in the miR-26b-5p inhibitor group. This also suggests that miR-26b-5p promotes osteogenesis and the opposite effect of FGF21. Furthermore, WB analysis showed that FGF21 expression in the miR-26b-5p overexpression group was significantly decreased, and increased FGF21 was found in the miR-26b-5p inhibition group. miR-26b-5p overexpression may reverse the suppression effect of FGF21 on osteogenic differentiation-associated protein expression levels; and miR-26b-5p overexpression may enhance the promotive effect of si-FGF21 on osteogenic differentiation-associated expression levels. These results provide evidence that miR-26b-5p influences BMSC’s osteogenic differentiation via suppressing FGF21.

A major limitation of the present study is our inability to further validate the regulatory effect of miR-26b-5p on osteocyte function after overexpression of the target gene FGF21.

## 6. Conclusions

miR-26b-5p can regulate the osteogenic differentiation of BMSCs and participate in PMOP pathogenesis via suppressing FGF21.

## Acknowledgements

All authors would like to thank to the key laboratory of basic pharmacology of Foshan Sanshui District People’s Hospital.

## Author contributions

**Conceptualization:** Bin Wang, Caiyuan Mai, Lei Pan.

**Data curation:** Bin Wang, Zhenhui Li, Caiyuan Mai, Lei Pan.

**Formal analysis:** Bin Wang.

**Funding acquisition:** Bin Wang.

**Investigation:** Bin Wang.

**Methodology:** Bin Wang, Zhenhui Li.

**Project administration:** Bin Wang.

**Resources:** Bin Wang, Penglin Mou.

**Software:** Bin Wang, Penglin Mou.

**Supervision:** Bin Wang, Caiyuan Mai, Penglin Mou.

**Validation:** Bin Wang, Zhenhui Li, Penglin Mou, Lei Pan.

**Visualization:** Bin Wang, Lei Pan.

**Writing – original draft:** Bin Wang, Caiyuan Mai.

**Writing – review & editing:** Bin Wang, Lei Pan.
